# Haptoglobin 2-2 Genotype Is Associated with TNF-**α** and IL-6 Levels in Subjects with Obesity

**DOI:** 10.1155/2014/912756

**Published:** 2014-04-29

**Authors:** Brissia Lazalde, Héctor M. Huerta-Guerrero, Luis E. Simental-Mendía, Martha Rodríguez-Morán, Fernando Guerrero-Romero

**Affiliations:** ^1^Biomedical Research Unit, Mexican Social Security Institute, Predio Canoas 100, Colonia Los Angeles, 34067 Durango, DGO, Mexico; ^2^Faculty of Medicine and Nutrition, Juárez University of Durango State, Avenida Universidad and Fanny Anitúa s/n, 34000 Durango, DGO, Mexico

## Abstract

*Objective*. To evaluate the association between Haptoglobin (HP) gene polymorphisms with inflammatory status in obese subjects. *Materials and Methods*. A cross-sectional study was carried out. A total of 276 apparently healthy men and nonpregnant obese women were enrolled and allocated according to the *HP* genotype into the *HP* ^1^/*HP* ^1^, *HP* ^2^/*HP* ^1^, and *HP* ^2^/*HP* ^2^ groups. Distribution of *HP* genotypes was 49, 87, and 140 for the *HP* ^1^/*HP* ^1^, *HP* ^2^/*HP* ^1^, and *HP* ^2^/*HP* ^2^, respectively. The *HP* genotype was determined using the polymerase chain reaction method. A multiple linear regression analysis adjusted by age, sex, waist circumference, and total body fat was used to determine the association between *HP* genotypes with TNF-**α**, IL-6, and high-sensitivity C-reactive protein (hsCRP) levels. *Results*. A multiple linear regression analysis adjusted by sex, waist circumference, and total body fat was performed showing a significant association between the *HP* ^2^/*HP* ^2^ genotype and TNF-**α** (**β** = 0.180; 95% CI 14.41–159.64, *P* = 0.01) and IL-6 (**β** = 0.188; 95% CI 1.53–12.72, *P* = 0.01) levels, but not with hsCRP (**β** = −0.008; 95% CI −1.64–1.47, *P* = 0.914) levels, whereas the *HP* ^2^/*HP* ^1^ genotype showed no association compared with the *HP* ^1^/*HP* ^1^ genotype (control group). *Conclusion*. Results of our study show that the *HP* ^2^/*HP* ^2^ genotype is associated with elevated TNF-**α** and IL-6, but not with hsCRP, levels in obese subjects.

## 1. Introduction

Obesity is associated with chronic low-grade inflammation through expansion of white adipose tissue (WAT), a lipid storage organ that secretes leptin, adiponectin, and adipokines such as interleukin (IL)-6, IL-1*β*, and TNF-*α* [1, 2]; in addition, hypertrophic adipocytes secrete chemoattractants such as monocyte chemoattractant protein (MCP-1) that promotes immune cells infiltration into WAT. Both events, chronic low-grade inflammation and infiltration of adipose tissue by macrophages, contribute to development of metabolic disorders [3, 4]. In this regard, although IL-6 and C-reactive protein (CRP) are well correlated with body mass index (BMI), TNF-*α* shows no correlation with neither BMI nor weight reduction [5].

The adipokine haptoglobin (HP), a plasmatic glycoprotein with tetrameric structure of 2 alpha and 2 beta polypeptides that are covalently binding by disulfide bonds [6], is a positive acute phase protein synthesized in the liver and expressed by WAT, exhibiting capacity to recruit monocytes and macrophages and thus playing an important role in the link between obesity and chronic systemic inflammation [7, 8].

In human, there are 3 common phenotypes of HP, Hp 1-1, Hp 2-2, and the heterozygous phenotype Hp 2-1. These phenotypes are controlled by two autosomal codominant alleles identified as* HP* ^1^ and* HP* ^2^ [9]. The* HP* ^2^ allele contains 1.7-kb intragenic duplication that arose after a unique nonhomologous DNA crossing-over within different introns of two* HP* ^1^ genes [10].

The HP protein, resulting from HP polymorphisms, has distinct biochemical and biophysical properties; it has been hypothesized that there is a greater expression of markers of activation in the macrophages of individuals with the HP 2-2 isoform as compared with individuals with HP 1-1 [11] that results in higher inflammatory status [8].

Thus, the aim of this study was to evaluate the association between* HP* gene polymorphisms with inflammatory status in obese subjects.

## 2. Materials and Methods

After protocol approval by the Mexican Social Security Institute Research Committee and after obtaining the written informed consent to participate, a cross-sectional study was carried out. Eligible subjects, apparently healthy obese men and nonpregnant obese women aged 18 to 65 years, were recruited from the general population of Durango, city in northern Mexico. According to the National Institute of Anthropology classification, all individuals were Mexican mestizos born in Mexico, had last name of Spanish origin, and had a family history of Mexican ancestry [12].

According* HP* genotypes, individuals were allocated into the* HP* ^1^ */HP* ^1^,* HP* ^2^ */HP* ^1^, and* HP* ^2^ */HP* ^2^ groups.

A body mass index (BMI) < 30 kg/m^2^, smoking, alcohol intake, acute or chronic inflammatory disease, acute or chronic infections, glomerulopathies, renal or hepatic disease, malignancy, and cardiovascular disease as well as intake of statins, fibrates, or anti-inflammatory drugs were exclusion criteria. A standardized interview, clinical examination, and laboratory tests were performed to carefully determine the presence of the inclusion and exclusion criteria.

### 2.1. Measurements

In the standing position and fasting conditions, weight, height, and waist circumference (WC) were measured with the subjects in light clothing and without shoes. Weight and height were measured using a fixed scale with stadimeter (Tanita TBF-215, Tokyo, Japan). The BMI was calculated as weight (kilograms) divided by height (meters) squared. Obesity was defined by BMI ≥ 30 kg/m^2^. The WC was measured to the nearest centimeter with a flexible steel tape; the anatomical landmarks used were midway between the lowest portion of the rib cage and the superior border of the iliac crest. Total body fat was measured by bioelectric impedance (Tanita TBF-215, Tokyo, Japan).

Blood pressure was measured according to recommendations of the Seventh Report of the Joint National Committee on Prevention, Detection, Evaluation, and Treatment of High Blood Pressure [13].

### 2.2. Assays

Whole blood sample was collected from antecubital venous under minimal tourniquet pressure, after 8–10 h overnight fasting. Serum biochemical determinations included plasma fasting glucose, total cholesterol, high-density lipoprotein cholesterol (HDL-c), low-density lipoprotein cholesterol (LDL-c), triglycerides, TNF-*α*, and IL-6.

Serum glucose was measured using the glucose-oxidase method; the intra- and interassay coefficients of variation were 1.1 and 1.5%. HDL-c fraction was obtained after precipitation by phosphotungstic reagent. Total cholesterol and triglycerides levels were enzymatically measured using spectrophotometric methods. The intra- and interassay coefficients of variation were 1.3% and 2.6% for HDL-c, 3.0% and 2.5% for total cholesterol, and 1.7% and 3.1% for triglycerides. All measurements were performed in an automatic chemical autoanalyzer (Data Pro Plus, Arlington Tx, USA).

TNF-*α* and IL-6 were measured using high sensitivity ELISA assays (Thermo Scientific kits, Rockford, IL), with range of 15.6–1000 pg/mL and 10.24–400 pg/mL, respectively, according to instructions provided by the manufacturer and evaluated using an iMark Microplate Absorbance Reader (Bio-Rad Laboratories Hercules, CA, USA).

Analysis of high-sensitivity C-reactive protein (hsCRP) was performed in a VITROS 5,1 FS Chemistry System (Ortho Clinical Diagnostics, Raritan, NJ, USA) based on particle-enhanced turbidimetry using the VITROS Chemistry Products (Johnson and Johnson Clinical Diagnostics Inc., Rochester, NY) with a detection limit of 0.32 mg/L and an extended measuring range of 0.32 mg/L to 42.83 mg/L (with auto-rerun), according to the manufacturer.

### 2.3. DNA Extraction and HP Genotyping

Genomic DNA was extracted from whole blood using DNAzol BD Reagent (Invitrogen, Carlsbad, CA). The* HP* genotype was determined using the polymerase chain reaction (PCR) method as previously described [14].

### 2.4. Statistical Analysis

The normality of the data and the homogeneity of  variances were tested using Shapiro-Wilk and Levene tests. When the data were normally distributed, the groups were compared using one-way ANOVA with post hoc Bonferroni test or a Chi-square test. When the distribution was not normal, particularly when homogeneity of variances was not observed, median values were compared using the Kruskal-Wallis and Mann-Whitney *U* test.

Given the nonparametric distribution of hsCRP levels, relationship between age and hsCRP levels was evaluated using the Spearman rank correlation test.

A multiple linear regression analysis was used to determine the association between HP genotypes (independent variable) with TNF-*α* and IL-6 levels (dependent variables). An additional multiple linear regression analysis, adjusted by age, sex, WC, and total body fat, was performed in order to control the influence on dependent variables.

The 95% confidence intervals (CI 95%) were determined, and a *P* value <0.05 defined statistical significance. Data were analyzed using the statistical package SPSS 15.0 (SPSS Inc., Chicago, IL, USA).

## 3. Results

A total of 328 subjects were screened; 52 (15.8%) individuals were excluded because they did not fulfill the inclusion criteria or by the presence of exclusion criteria. Thus, 276 (84.1%) individuals, 180 (65.2%) women and 96 (34.8%) men, with average age of 36.2 ± 12.5 years, were enrolled and allocated according to* HP* genotype into the* HP* ^1^ */HP* ^1^,* HP* ^2^ */HP* ^1^, and* HP* ^2^ */HP* ^2^ groups (Table 1).

Distribution of* HP* genotypes was 17.7%, 31.5%, and 50.7% for the* HP* ^1^ */HP* ^1^,* HP* ^2^ */HP* ^1^, and* HP* ^2^ */HP* ^2^, respectively. The genotype frequencies observed in this population showed a significant deviation from HWE (*P* < 0.05).

Table 1 shows the clinical and biochemical characteristics of the population in study; although subjects with* HP* ^2^ */HP* ^2^  genotype had lower WC than individuals with the* HP* ^1^ */HP* ^1^ genotype, they showed higher TNF-*α* levels; however, there were no significant differences in IL-6 and hsCRP levels between the groups.

In the overall population, age and hsCRP levels showed a significant positive relationship (*r* = 0.150, *P* = 0.02).

The unadjusted multiple linear regression analysis showed that* HP* ^2^ */HP* ^2^ genotype is significantly associated with TNF-*α* and IL-6, but not with hsCRP levels, whereas the* HP* ^2^ */HP* ^1^ genotype showed no association. In a subsequent multiple linear regression analysis adjusted by age, sex, WC, and total body fat the* HP* ^2^ */HP* ^2^ genotype remained significantly associated with TNF-*α* and IL-6 levels (Table 2).

A subanalysis comparing women and men who had* HP* ^2^ */HP* ^2^ genotype showed that WC, total body fat, triglycerides, and hsCRP, but not IL6 or TNF-*α* levels, were significantly higher in women than men (Table 3).

## 4. Discussion

Results of our study indicate that the* HP* ^2^ */HP* ^2^ genotype is associated with elevated TNF-*α* and IL-6 levels in obese subjects. However, in contrast with our finding, a recent study among Saudi diabetics and healthy subjects showed no association between HP phenotypes with serum IL-6 levels [15]; the inconsistency between our results and those by Mohieldein et al. [15] could be related to the target population and/or sample size.

Subjects with the* HP* ^2^ */HP* ^2^ genotype exhibited lower WC and higher levels of TNF-*α* and IL-6 than individuals with the* HP* ^1^ */HP* ^1^ genotype, finding that strongly suggests an increased capacity of type 2-2 to recruit macrophages in WAT, which are involved in the synthesis and release of TNF-*α* and IL-6 [16].

In addition, in our study, hsCRP levels were similar in the different groups included, findings in accordance with previous reports showing that there are no significant statistical differences in the hsCRP levels of apparently healthy individuals [17], patients with type 2 diabetes [14], and patients with peripheral occlusive disease [18], who exhibit phenotype Hp 2-2 compared with individuals exhibiting phenotypes Hp 1-1 and Hp 2-1. However, data are controversial with some studies showing that patients with chronic kidney disease [19] and those with essential hypertension [18] with phenotype Hp 2-2 have significantly higher levels of hsCRP than individuals with phenotypes Hp 1-1 and Hp 2-1. These inconsistencies have been explained based on genetic variations of* C-reactive protein* (CRP) gene, low levels of physical activity, and gradual increase of CRP with aging [14, 20]. Although in our population there was a positive relationship between hsCRP levels and age, we did not measure genetic variations of* CRP* gene nor physical activity.

Interestingly, among individuals with* HP* ^2^ */HP* ^2^ genotype, women showed significant higher hsCRP levels, but not differences between TNF-*α* and IL-6 levels than men; although differences could be related to the elevated total body fat and the high WC, further research is mandatory to elucidate the involved mechanisms in the association between* HP* genotypes and the triggering of low chronic systemic inflammation.

A model for the role that the HP plays in inflammation has been proposed; according to this model, through the IL-6 activity, the stressed cells emit warning signals triggering HP expression. In this way, among subjects with* HP* ^1^ */HP* ^1^ genotype, the HP significantly decreases synthesis of reactive oxygen species through its potent antioxidant function, whereas in the individuals with the* HP* ^2^ */HP* ^2^ genotype, the antioxidant activity is weak favoring the persistence of inflammatory response [21]. However, the effect of* HP* genotype in the inflammatory process is unclear and requires further research.

There is a worldwide variation in the frequency of alleles* HP* ^1^ and* HP* ^2^; in this regard, it has been proposed that* HP* ^2^ allele emerged in India and probably it was propagated due to a selective pressure, suggesting a selective advantage over the* HP* ^1^ allele [22].

Frequency of the* HP* ^2^ allele in our study was higher (66.4%) as compared with frequency previously reported in indigenous populations from Durango State (37.9%) [23], findings that could be explained in part because our study was focused on Mexican mestizos, from urban area of Durango city.

Several limitations of this study deserve to be mentioned. First, we did not measure serum levels of Hp; however, previously, no significant differences between* HP* genotypes and Hp phenotypes in healthy subjects with overweight or obesity were reported [15]; so, this limitation exerts minimal influence on our results and conclusion. Second, we did not include a control group of nonobese subjects; however, taking into account the aim of our study and the lack of consistency between TNF-*α* and BMI [5], which could introduce an analysis bias comparing individuals with different BMI, the noninclusion of a control group of normal weight individuals has no influence on our main conclusion. Third, population in study was not in HWE, which could be related to the target population that included only subjects with obesity.

## 5. Conclusion

In conclusion, our results show that the* HP* ^2^ */HP* ^2^ genotype is associated with elevated TNF-*α* and IL-6, but not with hsCRP, levels in obese subjects, suggesting that the functional differences of HP subtypes could be associated with different outcomes of obesity.

## Figures and Tables

**Table 1:** Characteristics of the study groups. *n* = 276.

	*HP* ^ 1^/*HP* ^1^	*HP* ^ 2^/*HP* ^1^	*HP* ^ 2^/*HP* ^2^	*P *
	*n* = 49	*n* = 87	*n* = 140	
Age, years	33.0 ± 11.7	37.1 ± 13.8	36.6 ± 11.6	0.155
Women, *n* (%)	22 (44.8%)	55 (63.2%)	103 (73.5%)	0.001**
Body mass index, kg/m^2^	34.6 ± 3.1	35.0 ± 4.9	35.0 ± 5.1	0.875
Waist circumference, cm	111.6 ± 9.4	109.8 ± 13.4	106.6 ± 11.1	0.01**
Total body fat, %	39.5 ± 6.3	42.1 ± 6.5	42.3 ± 6.4	0.02**
Systolic blood pressure, mmHg	118.4 ± 9.6	120.0 ± 14.6	117.5 ± 12.9	0.360
Diastolic blood pressure, mmHg	75.8 ± 8.4	76.9 ± 10.3	76.0 ± 8.7	0.719
Fasting glucose, mg/dL	104.7 ± 10.6	104.1 ± 14.8	106.0 ± 10.0	0.490
Total cholesterol, mg/dL	182.2 ± 39.9	187.6 ± 33.0	184.9 ± 42.4	0.730
HDL-cholesterol, mg/dL	38.5 ± 13.6	39.9 ± 13.9	38.3 ± 19.4	0.763
LDL-cholesterol, mg/dL	108.6 ± 40.2	111.4 ± 32.9	114.7 ± 47.8	0.244
Triglycerides, mg/dL*	154 (109–205)	163.0 (110–223)	137 (108–197)	0.261
TNF-*α*, pg/mL*	115.5 (66.5–232.7)	137.8 (91.3–202.2)	174.0 (91.9–298.5)	0.03^∗∗, †^
IL-6, pg/mL*	3.4 (1.0–6.5)	2.7 (1.0–8.2)	4.3 (1.0–9.5)	0.08
hsCRP, mg/L*	4.0 (1.8–6.5)	3.8 (1.6–8.4)	3.7 (2.2–8.6)	0.701

Values are mean ± standard deviation.

hsCRP: high-sensitivity C-reactive protein.

*P* value estimated using one-way ANOVA with post hoc Bonferroni test.

*Median (25–75th percentile), *P* value estimated using Kruskal-Wallis test.

**Statistical significant difference between *HP* ^1^/*HP* ^1^ and *HP* ^2^/*HP* ^2^.

^†^Statistical significant difference between *HP* ^2^/*HP* ^1^ and *HP* ^2^/*HP* ^2^.

**Table 2:** Multiple linear regression analysis adjusted by age, sex, waist circumference, and total body fat that evaluates the association between *HP* ^2^/*HP* ^2^ and *HP* ^2^/*HP* ^1^ genotypes (independent variables) with TNF-*α*, IL-6, and C-reactive protein (dependent variables). The *HP* ^1^/*HP* ^1^ was the control group.

	*HP* ^ 2^/*HP* ^2^ genotype			*HP* ^ 2^/*HP* ^1^ genotype			
	*β*	95% CI	*P *	*β*	95% CI	*P *
Model 1*						
TNF-*α*	0.154	6.26–142.16	0.03	0.026	−32.67–44.47	0.763
IL-6	0.142	0.04–10.69	0.04	0.142	−0.79–9.13	0.099
hsCRP	0.030	−1.25–1.93	0.676	0.044	−1.70–2.88	0.610

Model 2**						
TNF-*α*	0.180	14.41–159.64	0.01	0.048	−28.01–49.57	0.583
IL-6	0.188	1.53–12.72	0.01	0.171	−0.83–10.14	0.054
hsCRP	−0.008	−1.64–1.47	0.914	−0.018	−2.46–1.99	0.834

hsCRP: high-sensitivity C-reactive protein.

*Unadjusted.

**Adjusted by age, sex, waist circumference, and total body fat.

**Table 3:** Characteristics of the *HP* ^2^/*HP* ^2^ genotype according to sex. *n* = 140.

	*HP* ^ 2^/*HP* ^2^		*P *
	Women *n* = 103	Men *n* = 37	
Age, years	37.3 ± 11.3	34.5 ± 12.3	0.229
Body mass index, kg/m^2^	35.0 ± 5.5	35.0 ± 3.8	0.992
Waist circumference, cm	103.8 ± 10.2	114.5 ± 9.7	<0.001
Total body fat, %	44.6 ± 4.6	35.8 ± 6.5	<0.001
Systolic blood pressure, mmHg	115.8 ± 13.1	122.0 ± 11.2	0.008
Diastolic blood pressure, mmHg	75.3 ± 8.3	77.8 ± 9.6	0.172
Fasting glucose, mg/dL	106.7 ± 10.2	104.0 ± 9.2	0.148
Total cholesterol, mg/dL	184.3 ± 43.1	186.5 ± 41.0	0.778
HDL-cholesterol, mg/dL	37.2 ± 8.9	41.2 ± 35.0	0.498
LDL-cholesterol, mg/dL	119.0 ± 41.0	102.8 ± 62.2	0.146
Triglycerides, mg/dL*	128 (100–168)	192 (143–252)	<0.001
TNF-*α*, pg/mL*	156.5 (90.1–301.8)	216.4 (99.9–314.8)	0.502
IL-6, pg/mL*	4.3 (1.0–8.7)	4.8 (1.0–10.8)	0.983
hsCRP, mg/L*	4.2 (2.7–8.9)	2.2 (1.0–8.5)	0.007

Values are mean ± standard deviation.

hsCRP: high-sensitivity C-reactive protein.

*Median (25–75th percentile), *P* value estimated using Mann-Whitney *U* test.

## References

[1] Hotamisligil GS (2006). Inflammation and metabolic disorders. *Nature*.

[2] Browning LM, Krebs JD, Magee EC, Frühbeck G, Jebb SA (2008). Circulating markers of inflammation and their link to indices of adiposity. *Obesity Facts*.

[3] Fantuzzi G (2005). Adipose tissue, adipokines, and inflammation. *Journal of Allergy and Clinical Immunology*.

[4] Osborn O, Olefsky JM (2012). The cellular and signaling networks linking the immune system and metabolism in disease. *Nature Medicine*.

[5] Browning LM, Krebs JD, Magee EC, Frühbeck G, Jebb SA (2008). Circulating markers of inflammation and their link to indices of adiposity. *Obesity Facts*.

[6] Yang F, Brune JL, Baldwin WD (1983). Identification and characterization of human haptoglobin cDNA. *Proceedings of the National Academy of Sciences of the United States of America*.

[7] Friedrichs WE, Navarijo-Ashbaugh AL, Bowman BH, Yang F (1995). Expression and inflammatory regulation of haptoglobin gene in adipocytes. *Biochemical and Biophysical Research Communications*.

[8] Maffei M, Funicello M, Vottari T (2009). The obesity and inflammatory marker haptoglobin attracts monocytes via interaction with chemokine (C-C motif) receptor 2 (CCR2). *BMC Biology*.

[9] Smithies O, Walker NF (1956). Notation for serum-protein groups and the genes controlling their inheritance. *Nature*.

[10] Maeda N, Yang F, Barnett DR (1984). Duplication within the haptoglobin Hp2 gene. *Nature*.

[11] Nakhoul FM, Miller-Lotan R, Awaad H, Asleh R, Levy AP (2007). Hypothesis—haptoglobin genotype and diabetic nephropathy. *Nature Clinical Practice Nephrology*.

[12] Gorodezky C, Alaez C, Vázquez-García MN (2001). The genetic structure of Mexican Mestizos of different locations: tracking back their origins through MHC genes, blood group systems, and microsatellites. *Human Immunology*.

[13] Chobanian AV, Bakris GL, Black HR (2003). Seventh report of the joint national committee on prevention, detection, evaluation, and treatment of high blood pressure. *Hypertension*.

[14] Koch W, Latz W, Eichinger M (2002). Genotyping of the common haptoglobin Hp 1/2 polymorphism based on PCR. *Clinical Chemistry*.

[15] Mohieldein A, Alzohairy M, Hasan M, Khan AA (2012). Inflammatory markers and haptoglobin polymorphism in Saudi with non-insulin-dependent diabetes mellitus. *Global Journal of Health Science*.

[16] Weisberg SP, McCann D, Desai M, Rosenbaum M, Leibel RL, Ferrante AW (2003). Obesity is associated with macrophage accumulation in adipose tissue. *Journal of Clinical Investigation*.

[17] Braeckman L, de Bacquer D, Delanghe J, Claeys L, de Backer G (1999). Associations between haptoglobin polymorphism, lipids, lipoproteins and inflammatory variables. *Atherosclerosis*.

[18] Delanghe JR, Duprez DA, de Buyzere ML (1993). Haptoglobin polymorphism and complications in established essential arterial hypertension. *Journal of Hypertension*.

[19] Strandhave C, Svensson M, Krarup H, Christensen JH (2013). Haptoglobin genotype and risk markers of cardiovascular disease in patients with chronic kidney disease. *International Journal of Nephrology*.

[20] Kluft C, de Maat MPM (2003). Genetics of C-reactive protein: new possibilities and complications. *Arteriosclerosis, Thrombosis, and Vascular Biology*.

[21] Quaye IK (2008). Haptoglobin, inflammation and disease. *Transactions of the Royal Society of Tropical Medicine and Hygiene*.

[22] Langlois MR, Delanghe JR (1996). Biological and clinical significance of haptoglobin polymorphism in humans. *Clinical Chemistry*.

[23] Delanghe J, Langlois M, Alvarado Esquivel C, de Haene H, de Buyzere M (2000). Haptoglobin 1F allele frequency is high among indigenous populations in the state of Durango, Mexico. *Human Heredity*.

